# Prevalence and survival prognosis of prostate cancer in patients with end-stage renal disease: a retrospective study based on the Korea national database (2003–2010)

**DOI:** 10.18632/oncotarget.19453

**Published:** 2017-07-22

**Authors:** Sung Han Kim, Jae Young Joung, Yoon Seok Suh, Young Ae Kim, Jin Hyuk Hong, Tong Sun Kuark, Eun Sook Lee, Kang Hyun Lee

**Affiliations:** ^1^ Department of Urology, Center for Prostate Cancer, Research Institute and Hospital of National Cancer Center, Goyang, Korea; ^2^ Cancer Policy Branch, National Cancer Control Institute, National Cancer Center, Goyang, Korea; ^3^ Health Insurance Policy Research Institute, National Health Insurance Service, Seoul, Korea; ^4^ Center for Breast Cancer, Research Institute and Hospital, National Cancer Center, Goyang, Korea

**Keywords:** prostate cancer, end-stage renal disease, transplantation, prostate-specific antigen, prognosis

## Abstract

**Objective:**

The study was aimed to evaluate the prevalence and prognosis of prostate cancer (PC) and end-stage renal disease (ESRD), determine the risk factors for overall survival (OS) and PC-specific survival (CSS), and evaluate differences in PC-related clinical therapeutic patterns between patients with and without PC-ESRD.

**Methods:**

This observational population study, performed at the National Cancer Center and Cancer Research Institute in Korea, included patients with PC and ESRD from the nationwide Korean Health Insurance System and Korean Central Cancer Registry data. Five-year overall and cancer-specific survival. A joinpoint regression analysis was performed to predict incidence and mortality of PC. Survival was analyzed using Kaplan-Meir curves with log rank tests of patients with dialysis or transplantation.

**Results:**

Of 3945 patients with PC-ESRD, 3.9% were on dialysis (N=152), 0.2% had kidney transplantation (N=10, D-TPL group); 3783 (95.9%) had neither dialysis nor transplantation (non-D-TPL ESRD group). There were 697 PC-specific deaths. The median respective OS, PC-specific survival, and 5-year survival rates in the non-ESRD, non-D-TPL ESRD, dialysis ESRD, and transplantation ESRD groups were significantly different (p<0.001). Presence of ESRD, age, body mass index, SEER stage, no treatment within 6 months after diagnosis, no surgery, chemotherapy, radiotherapy or hormonal therapy, non-adenocarcinoma pathology, and Charlson comorbidity index were independent risk factors for OS and CSS.

**Conclusions:**

With a 10.1% nationwide prevalence of PC-ESRD, the presence of ESRD was a significant survival factor along with other significant clinicopathological factors.

## INTRODUCTION

Globally, recent medical improvements have prolonged overall survival (OS) in all disease fields, including end-stage renal disease (ESRD) [1–2]. The enhanced surgical techniques and modern immunosuppression in renal transplantation have led to significant improvements in OS and graft survival, with approximately 35.9 years in the transplant half-life of grafts from living donors and 19.5 years for cadaveric grafts [3]. However, despite the necessity of renal transplantation for ESRD, preconditions for organ recipients are multifactorial because of the limited number of available kidneys. One of the conditions for kidney transplantation candidacy is the confirmation of a solid-organ malignancy-free state. To maximize the allocation of resources, the life expectancy of recipients should not be substantially lower than the life of their graft.

Over the past 10 and 15 years, the frequency of recipients older than 50 years has increased by 13% and 21%, respectively [1, 3–6]. Intuitively, this older male population is at a higher risk for prostate cancer (PC). Additionally, the increasing age of kidney transplant recipients, along with the increased age at initiation of dialysis and the documented increase in survival of patients with ESRD with or without dialysis after kidney transplant, has led to a higher number of kidney transplant recipients diagnosed with PC (1.4–5%) [7] at a two- to five-fold higher incidence than that for the general population [2, 6, 8]. Previous cancer statistics reported that PC is the second most prevalent solid malignancy in transplantation as well as the most common solid malignancy and the second leading cause of cancer death among American men [9]. Thus, the potential for developing PC has become an important clinical concern because of its morbidity and mortality, particularly because of the increase in number of elderly patients undergoing dialysis and renal transplantation. Additionally, PC-related issues associated with the guidelines for prostate-specific antigen (PSA) screening and standard active modalities of care for patients with ESRD diagnosed with PC (PC-ESRD) have emerged as one of the major issues of debate [5, 10].

Disagreements on the screening and treatment guidelines for patients with PC-ESRD have resulted from the lack of information on ESRD in combination with PC. Therefore, it is important to evaluate the clinico-pathological and prognostic characteristics of PC-ESRD, and to analyze the risk factors for OS and PC-specific survival (CSS). No previous studies have reported Asian patients with PC-ESRD, except for one on the epidemiology after transplantation in Taiwan [11]. Therefore, the aims of the present study were to evaluate the prevalence and prognosis of PC-ESRD in Korea, to determine the risk factors for OS and CSS, and to evaluate differences in PC-related clinical therapeutic patterns between patients with and without PC-ESRD.

## RESULTS

**Figure 1:**
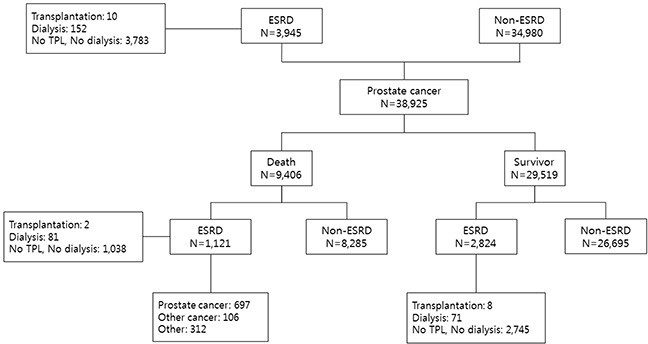
Study flow chart ESRD, end-stage renal disease; TPL, transplantation;ï»¿ Ca, cancer.

The study flow chart is presented in Figure 1. Compared to the non-ESRD groups, the ESRD group was significantly older, with a higher frequency of obese individuals, had an increasingly higher rate of PC diagnoses over time, had more localized PC, was less actively, surgically, and radio-therapeutically treated, had more non-adenocarcinoma histopathology, had a higher grade of CGI, and a higher non-PC-related death rate (all p<0.001; Table 1). Overall, 29,519 (75.8%) patients were alive at the start of study period, with a median survival time of 37.4 months (0-108.1 months); of these, 2824 (9.6%) patients with ESRD had a median survival time of 29.9 months (0-108.1 months, Table 1). Among 9406 (24.2%) deaths, 1121 (11.9%) patients with ESRD died due to PC-specific deaths (7.4%, N=697) with a median survival of 2.9 years (Figure 1).

**Table 1:** Demographics of patients in the ESRD and non-ESRD groups

Parameter	ESRD (N=3,945)		Non-ESRD (N=34,980)		P-value
	N	%	N	%	
**Age (N, %)**					
<55	95	2.41	1,503	4.30	<0.001
55-65	735	18.63	8,897	25.43	
65-75	1,914	48.52	16,082	45.97	
75<	1,201	30.44	8,496	24.29	
**BMI** (kg/m^2^)					
Underweight (<18.5)	62	2.54	741	3.30	<0.001
Normal (18.5∼22)	779	31.86	7,941	35.33	
Overweight (23∼24)	699	28.59	6,391	28.43	
Obesity (25∼)	905	37.01	7,403	32.94	
Missing					
**Year of cancer diagnosis**					
2003∼2004	354	8.97	5,353	15.30	<0.001
2005∼2007	1,188	30.11	11,706	33.46	
2008∼2010	2,403	60.91	17,921	51.23	
**SEER stage**					
Localized	1,974	54.97	15,572	52.56	<0.001
Regional nodes, both, NOS	555	15.46	5,579	18.83	
Distant	324	9.02	2,722	9.19	
Unknown	738	20.55	5,754	19.42	
**Treatment within 6-month after cancer diagnosis**					
Yes	3,234	81.98	29,632	84.71	<0.001
No	711	18.02	5,348	15.29	
**Surgery treatment**					
Prostatectomy	2,120	53.74	20,486	58.56	<0.001
Other	1,825	46.26	14,494	41.44	
**Chemo treatment**					
Yes	780	19.77	6,952	19.87	0.8786
No	3,165	80.23	28,028	80.13	
**Radiation treatment**					
Yes	674	17.08	6,870	19.64	<0.001
No	3,271	82.92	28,110	80.36	
**Hormonal treatment**					
Yes	2,024	51.31	18,443	52.72	0.0906
No	1,921	48.69	16,537	47.28	
**Type of pathology**					
Adenocarcinoma	3,063	77.64	28,011	80.08	0.0003
Others	882	22.36	6,969	19.92	
**CCI group**					
0	166	4.21	4,848	13.86	<0.001
1	506	12.83	8,304	23.74	
2	3,273	82.97	21,828	62.40	
**Death**					
No	2,824	71.58	26,695	76.32	<0.001
Yes	1,121	28.42	8,285	23.68	
**Cause of death**					
Prostate cancer	697	62.51	5,439	66.02	0.0001
Other cancer	106	9.51	944	11.46	
Other	312	27.98	1,856	22.53	
Survival time*	29.9	0-108.0	37.4	0-108.1	<0.001

* Median (range) months.

In the 3945 patients with ESRD, differences in age, body mass index (BMI), time since cancer diagnosis, chemotherapy rate, radiotherapy rate, and treatment rate within 6 months after diagnosis were not significant between the patients without dialysis and transplantation (ND-TPL group) and patients with dialysis or kidney transplantation (D-TPL group). However, compared to the ND-TPL group, the D-TPL group had a significantly higher rate of advanced stage PC, hormonal therapy, and death, while, surgical therapy was performed less frequently in this group (p<0.001, Table 2). The median survival times between the D-TPL and ND-TPL groups were 20.2 months (0-107.1 months) and 30.5 months (0-108.0 months), respectively (p=0<0.001, Table 2).

**Table 2:** Demographics of patients in the D-TPL and ND-TPL groups among those with ESRD

Parameter	ND-TPL (N=3,783)		D-TPL (N=162)		P-value
	N	%	N	%	
**Age**					
<55	92	2.4	3	1.9	0.7723
55-65	706	18.7	29	17.9	
65-75	1,839	48.6	75	46.3	
75<	1,146	30.3	55	34.0	
**BMI** (kg/m^2^)					
Underweight (<18.5)	58	2.4	4	5.9	0.0728
Normal (18.5∼22)	751	31.6	28	41.2	
Overweight (23∼24)	685	28.8	14	20.6	
Obesity (25∼)	883	37.2	22	32.4	
**Year of cancer diagnosis**					
2003∼2004	331	8.8	23	14.2	0.0557
2005∼2007	1,140	30.1	48	29.6	
2008∼2010	2,312	61.1	91	56.2	
**SEER stage**					
Localized	1,922	55.7	52	37.4	<0.001
Regional extension only, lymphnodes, both, NOS	537	15.6	18	13.0	
Distant	296	8.6	28	20.1	
Unknown	697	20.2	41	29.5	
**Treatment within 6-month after cancer diagnosis**					
Yes	3,110	82.2	124	76.5	0.0661
No	673	17.8	38	23.5	
**Surgery treatment**					
Prostatectomy	2,065	54.6	55	34.0	<0.001
Other	1,718	45.4	107	66.1	
**Chemo treatment**					
Yes	744	19.7	36	22.2	0.4239
No	3,039	80.3	126	77.8	
**Radiation treatment**					
Yes	654	17.3	20	12.4	0.1017
No	3,129	82.7	142	87.7	
**Hormonal treatment**					
Yes	1,924	50.9	100	61.7	0.0067
No	1,859	49.1	62	38.3	
**Type of pathology**					
Adenocarcinoma	2,946	77.9	117	72.2	0.0908
Others	837	22.1	45	27.8	
**CCI group**					
0	150	4.0	16	9.9	0.0007
1	482	12.7	24	14.8	
2=<	3,151	83.3	122	75.3	
**Death**					
No	2,745	72.6	79	48.8	<0.001
Yes	1,038	27.4	83	51.2	
**Cause of death**					
Cancer (prostate)	649	62.9	48	57.8	0.1658
Other cancer	101	9.8	5	6.0	
Other	282	27.3	30	36.1	
Survival time*	30.5	0-108.0	20.2	0-107.1	<0.001

* Median (range) months.

**Figure 2:**
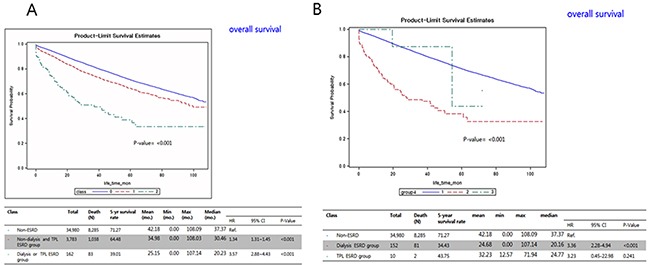
Kaplan-Meier overall survival curves comparing the **(A)** non-ESRD, ESRD without dialysis and transplantation, and ESRD with dialysis and transplantation groups; and the **(B)** ESRD without dialysis and transplantation, and ESRD with dialysis and transplantation groups. ESRD, end-stage renal disease; TPL, transplantation; CI, confidence interval; mo., month; HR, hazard ratio.

**Figure 3:**
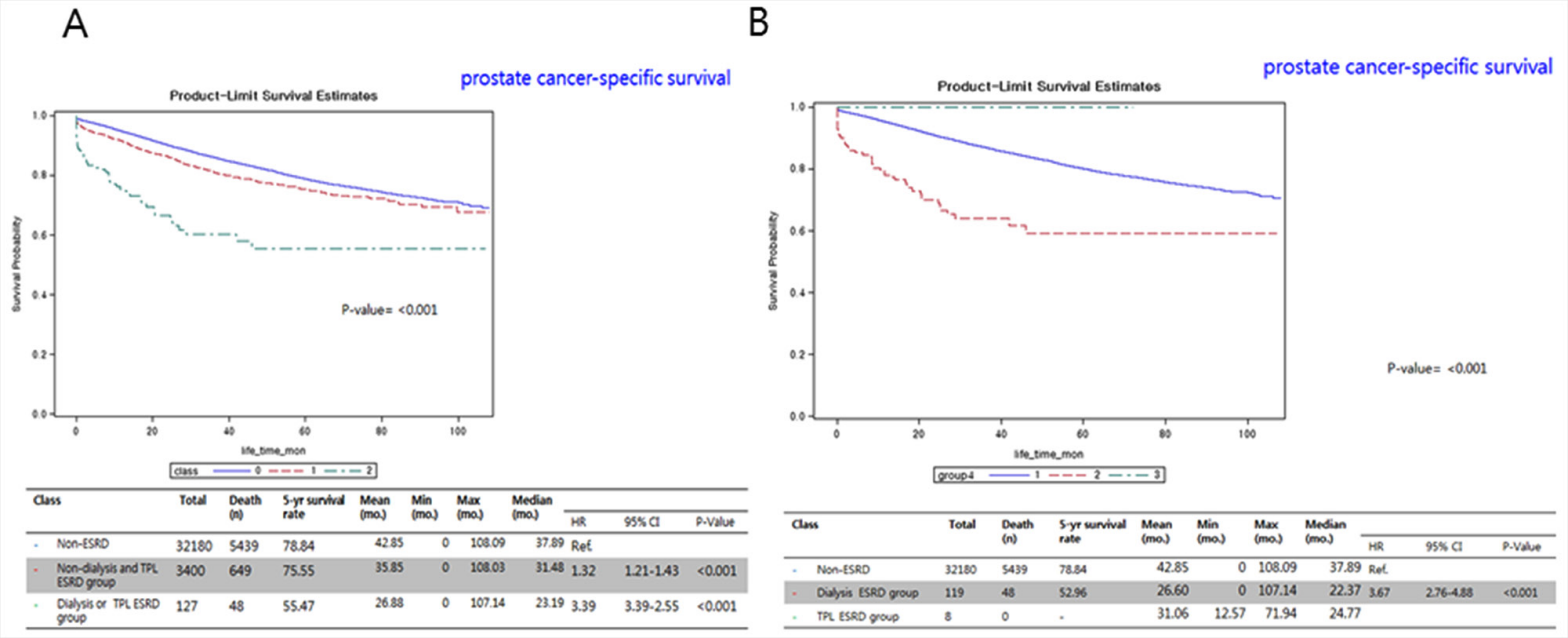
Kaplan-Meier prostate cancer-specific survival curves between the **(A)** non-ESRD, ESRD without dialysis and transplantation, and ESRD with dialysis and transplantation groups; and **(B)** non-ESRD, dialysis-ESRD, and TPL-ESRD groups. ESRD, end-stage renal disease; TPL, transplantation; CI, confidence interval; mo., month; HR, hazard ratio.

Results of the comparative 5-year OS rate curves showed significant differences between the non-ESRD and ND-TPL groups (71.3% vs. 64.5%, hazard ratio [HR] 1.34, 95% confidence interval [CI] 1.31–1.45), and between the non-ESRD and D-TPL groups (71.3% vs. 39.0%, HR 3.57, 95% CI 2.88–4.43; p<0.001; Figure 2). The cancer-specific survival curve showed a significantly similar pattern to the OS curves, as compared to the non-ESRD group (78.8%), the ND-TPL (75.6%), and D-TPL (55.5%) groups had lower 5-year CSS rates (p<0.001, Figure 3).

Results of the adjusted multivariate analysis showed that being in the ESRD group, age, BMI, SEER stage, no treatment within 6 months after diagnosis, prostatectomy, no chemotherapy, no radiation, no hormonal therapy, a non-adenocarcinoma pathology, and CCI group were significant risk factors for OS (Table 3). Further multivariate analysis for prostate-CSS with competing-risks analysis, the ESRD group, age, BMI, SEER stage, no treatment within 6 months after diagnosis, prostatectomy, no chemotherapy, no radiation, no hormonal therapy, a non-adenocarcinoma pathology, and CCI group were significant risk factors for CSS (Table 4).

**Table 3:** Results of multivariate analysis for overall survival

Parameter	Crude analysis				Adjusted analysis			
	Hazard ratio	95% confidence interval		P-value	Hazard ratio	95% confidence interval		P-value
		Lower limit	Upper limit			Lower limit	Upper limit	
**Group**								
Non-ESRD (n=34,980)	REF.				REF.			
ND-TPL (n=3,783)	1.394	1.307	1.487	<0.001	1.564	1.411	1.734	<0.0001
D-TPL (n=162)	3.571	2.877	4.434	<0.001	3.226	2.206	4.716	<0.0001
**Age**								
<55	REF.				REF.			
55-65	0.997	0.857	1.161	0.974	1.298	1.004	1.679	0.0466
65-75	1.665	1.441	1.925	<0.001	2.085	1.629	2.669	<0.0001
75<	4.732	4.097	5.466	<0.001	4.401	3.428	5.651	<0.0001
**BMI** (kg/m^2^)								
Underweight <18.5)	REF.				REF.			
Normal (18.5∼22)	1.882	1.663	2.131	<0.001	1.428	1.241	1.644	<0.0001
Overweight (23∼24)	0.681	0.633	0.734	<0.001	0.775	0.713	0.843	<0.0001
Obesity (25∼)	0.618	0.575	0.665	<0.001	0.778	0.717	0.845	<0.0001
**SEER stage**								
Localized	REF.				REF.			
Regional NOS	1.141	1.053	1.237	0.001	1.276	1.146	1.421	<0.0001
Distant	6.711	6.300	7.150	<0.001	5.044	4.609	5.520	<0.0001
Unknown	2.355	2.214	2.506	<0.001	1.827	1.674	1.993	<0.0001
**Active treatment within 6 months after diagnosis**	REF.				REF.			
None	1.315	1.243	1.390	<0.001	2.318	2.009	2.675	<0.0001
**Treatment**								
Prostatectomy	2.861	2.744	2.984	<0.001	1.436	1.331	1.549	<0.0001
No chemo Tx	0.888	0.845	0.933	<0.001	0.750	0.693	0.811	<0.0001
No radiation Tx	0.927	0.883	0.973	<0.001	0.749	0.691	0.811	<0.0001
No hormonal Tx	0.428	0.410	0.448	<0.001	0.465	0.417	0.519	<0.0001
**Non-adenocarcinoma pathology CCI group**	1.662	1.587	1.740	<0.001	1.363	1.262	1.473	<0.0001
0	REF.				REF.			
1	0.598	0.562	0.636	<0.001	0.710	0.636	0.792	<0.0001
2	0.477	0.453	0.503	<0.001	0.602	0.546	0.664	<0.0001

**Table 4:** Results of multivariate analysis for prostate cancer-specific survival

Parameter	Crude analysis				Adjusted analysis				Adjusted analysis*			
	Hazard ratio	95% confidence interval		P-value	Hazard ratio	95% confidence interval		P-value	Hazard ratio	95% confidence interval		P-value
		Lower limit	Upper limit			Lower limit	Upper limit			Lower limit	Upper limit	
**Group**												
Non-ESRD (n=34,980)	REF.				REF.				REF.			
ND-TPL (n=3,783)	1.298	1.298	1.408	<0.001	1.573	1.380	1.794	<0.0001	1.488	1.295	1.708	<0.0001
D-TPL (n=162)	3.021	2.274	4.014	<0.001	2.503	1.478	4.238	0.0006	2.050	1.172	3.588	0.0119
**Age**												
<55	REF.				REF.				REF.			
55-65	0.879	0.744	1.037	0.1269	1.167	0.884	1.541	0.2745	1.162	0.898	1.504	0.2535
65-75	1.232	1.052	1.444	0.0098	1.604	1.229	2.093	0.0005	1.564	1.223	2.002	0.0004
75<	3.483	2.976	4.078	<0.001	3.285	2.507	4.305	<0.0001	3.024	2.346	3.897	<0.0001
**BMI** (kg/m^2^)												
Underweight <18.5)	REF.				REF.				REF.			
Normal (18.5∼22)	1.874	1.607	2.186	<0.001	1.519	1.280	1.802	<0.0001	1.492	1.234	1.802	<0.0001
Overweight (23∼24)	0.671	0.611	0.736	<0.001	0.762	0.686	0.847	<0.0001	0.776	0.696	0.865	<0.0001
Obesity (25∼)	0.618	0.564	0.676	<0.001	0.773	0.699	0.856	<0.0001	0.790	0.712	0.876	<0.0001
**SEER stage**												
Localized	REF.				REF.				REF.			
Regional NOS	1.401	1.261	1.557	0.001	1.509	1.308	1.741	<0.0001	1.489	1.292	1.717	<0.0001
Distant	11.234	10.387	12.150	<0.001	7.922	7.075	8.870	<0.0001	7.626	6.772	8.587	<0.0001
Unknown	3.081	2.839	3.344	<0.001	2.314	2.058	2.601	<0.0001	2.298	2.041	2.587	<0.0001
**Active treatment within 6 months after diagnosis, Yes**	REF.				REF.				REF.			
None	1.299	1.212	1.392	<0.001	3.464	2.837	4.229	<0.0001	3.264	2.690	3.959	<0.0001
**Treatment**												
Prostatectomy	3.150	2.988	3.321	<0.001	1.443	1.315	1.584	<0.0001	1.410	1.279	1.554	<0.0001
No chemo Tx	1.010	0.948	1.077	0.7494	0.846	0.764	0.937	0.0013	0.878	0.790	0.977	0.0166
No radiation Tx	0.749	0.708	0.793	<0.001	0.630	0.572	0.693	<0.0001	0.610	0.553	0.673	<0.0001
No hormonal Tx	0.341	0.322	0.361	<0.001	0.312	0.265	0.367	<0.0001	0.313	0.265	0.370	<0.0001
**Non-adenocarcinoma pathology CCI group**	1.805	1.708	1.908	<0.001	1.428	1.300	1.569	<0.0001	1.387	1.257	1.530	<0.0001
0	REF.				REF.				REF.			
1	0.541	0.504	0.581	<0.001	0.667	0.587	0.757	<0.0001	0.679	0.594	0.776	<0.0001
2	0.361	0.339	0.384	<0.001	0.502	0.448	0.563	<0.0001	0.512	0.453	0.578	<0.0001

* Competing-risks analysis.

## DISCUSSION

Studies that have examined the risk and prognosis of PC diagnosed in the cohort of ESRD, particularly transplanted cohorts, are difficult to interpret; in addition, the history of untreated patients with PC-ESRD is not well characterized because of the detection bias introduced by more intensive and routine screening. However, the United States Preventive Services Task Force suggested PSA screening as a grade D recommendation [12]. Additionally, many recent studies have suggested that PSA screening in cases of ESRD is unnecessary due to the increased over diagnosis of clinically insignificant PC, which may not impact survival without treatment, or the common course of chronic immunsuppression after transplantation [7, 13–15]. Similar to our finding of a 54.0% localized PC rate in the ESRD group according to the SEER stages (Table 1), most patients with PC-ESRD have localized PC (53–87.4%) with a low grade (91% cT1c, 72% Gleason sum 6, and 100% pT2 at prostatectomy) and excellent outcomes after treatment (100% recurrence-free survival) [5, 16]. Therefore, PC does not affect the overall mortality, but renal failure is the primary survival-determining factor in ESRD; thus, kidney transplantation is the most important survival factor in patients with ESRD [7, 12–15].

As one of the pre-requisite conditions for kidney transplantation is a solid-organ malignancy-free state, patients with PC-ESRD and a positive PSA screening test are ineligible for receiving a kidney transplant until a disease-free state is confirmed; thus, their transplantation priority is delayed. A positive screening result for PC significantly increases transplant wait times by about 2–3.5 fold, especially in ESRD patients less than 69 years old, and 75.8% of candidates with ESRD and a positive PSA screening result never received a transplant [7]. In the clinical settings, most clinicians (89%) from 195 United States transplantation centers conducted their routine PSA screening in patients with ESRD, and 73% of them waited until the patients met the eligibility criteria after treatment [17]. In the present study, a low rate of transplant patients with PC-ESRD (3.3%, N=13) and a poor CSS time in patients with PC-ESRD on dialysis (29.4 months vs. 24.8 months [TPL-PC-ESRD] vs. 20.2 months [ND-TPL PC-ESRD]), indirectly implied that patients with PC-ESRD had longer wait times and fewer opportunities of receiving kidney transplants until they achieved a cancer-free state due to the present shortage of grafts (p<0.01, Table 1; Figure 2).

The results of our predictive risk analysis showed that D-TPL ESRD and ND-TPL ESRD were significant independent factors of OS (Table 3). The results of multivariate analysis for prostate CSS with competing-risks analysis showed that D-TPL ESRD and ND-TPL ESRD were significant independent factors of CSS (Table 4). The D-TPL ESRD group had a significantly higher rate of advanced stage PC (62.6%) and a worse OS (20.2 months) than the ND-TPL ESRD group (30.5 months), which had an advanced PC rate of 44.3% (p<0.001, Tables 1 and 2; Figures 2 and 3). The CSS of patients with PC-ESRD who underwent transplantation (24.7 months) was similar to that in the non-PC-ESRD group (37.9 months) (Figure 3B); thus, transplanted ESRD patients may be considered as non-ESRD. The American Society of Transplantation recommends physical screening after renal transplantation using a digital rectal examination and PSA screening starting at the age of 50 years in men with a life expectancy of >10 years [18]. A recent survey of 195 renal transplantation centers in United States on the practical treatment and screening of PSA in renal transplant patients reported that most clinicians (89%) perform routine PSA screening in patients with ESRD using their specific guidelines [17].

Standard treatments can be performed in transplant patients with PC-ESRD ensuing satisfactory results in terms of both the oncological outcomes and graft function, which would be similar to those reported in previous studies. D-TPL ESRD patients and non-D-TPL PC-ESRD patients who are candidates for transplantation and have a survival expectancy of 10 years, should be screened for the risks of PC using active PSA screening, because the survival of these patients is potentially the same as that of the general population. The survival of patients with ESRD should be further analyzed, to stratify patients who benefit from PSA screening. Thus, ESRD patients undergoing dialysis, with a <10-year expected survival are not necessarily candidates for PC screening.

An active PC screening, especially in dialysis and transplant patients with ESRD is based on the fact that patients with PC-ESRD usually presented at a more advanced clinical stage and a grade ≥2 (47–55.3% and 45%, respectively, in our study). Moreover, they had an increased tumor volume, shown by the percentage of positive biopsy scores, rapid malignant cell proliferation, aggressive biochemical behavior, and a higher susceptibility to infections [8, 19, 20]. Our study also showed that 62.6% of transplanted PC-ESRD patients were diagnosed with locally advanced or advanced PC. In addition, the required immunosuppression after renal allograft transplant may hinder the immune surveillance of PC because of higher susceptibility to tumorigenesis and infections due to an increased incidence of solid-organ malignancy, suggesting a more aggressive disease course. Forty-seven percent of transplant recipients newly diagnosed with PC-ESRD at a minimum of 1 year after transplantation presented with a locally advanced lesion (T3 or T4) [8], and transplanted PC-ESRD recipients with active PC treatment achieved a longer survival, which may increase PC-specific mortality over an extended period [21].

Regarding the PSA references for biopsies and treatment of PC-ESRD, no established PSA ranges with an age reference to ESRD have been studied to determine susceptible patients with positive PC biopsies compared to the general population [22, 23]. Additionally, surgery and other curative forms of treatment such as radiation are associated with risks of damaging the allograft after transplantation. Patients with ESRD have higher PSA levels than the general population [22], and prostatectomy after transplantation is technically challenging [24, 25]. Radical prostatectomy is a good treatment option for ESRD before transplantation, because it enables patients with ESRD to achieve objective evidence of biochemical control rapidly post-surgery, which makes them eligible for renal transplantation (disease-free state). However, recent small-scale studies have reported successful treatment outcomes using all treatment modalities, including robotic surgeries [21, 26]. Our study also showed that most of the PC-ESRD patients, especially transplant and dialysis patients, were less frequently treated with surgery than other treatment modalities (p<0.05, Tables 1 and 2). Therefore, standard treatments could be proposed for transplant patients with PC-ESRD to achieve satisfactory results in terms of both, the oncological outcomes and graft function, which is consistent with previous reports.

As with all observational population-based studies, the present study has several limitations. First, although we adjusted for various potential covariates and ICD codes, variables such as lifestyle factors that affect ESRD were not included or routinely captured in a National Institutes of Health (NIH) database, because the database did not capture patients outside of the NIH system. Second, the medical therapies and ESRD patients with failed renal transplantation were not evaluated in terms of survival. Third, any information on the nuclear grades of PC was not obtained, which is important for determining the aggressiveness of PC. Fourth, because of fundamental limitations of this study using huge data with unavailability of clinico-pathological characteristics of patients, including the severity of the disease and tumor burden status in this study. Further prospective studies are needed to verify the poorer survival outcome in, both, OS and CSS in patients with radical prostatectomy found in this study. All the significant prognostic survival factors (OS and CSS), such as Gleason score, treatment interval time after diagnosis, and non-adenocarcinoma pathology were not evaluated in this study because of their unavailability in the National health database.

Despite these limitations, our study offers additional insights into PC-ESRD by extending the evidence to a nationwide cohort, for first time showing the clinical practice trends of diagnosing and treating patients with PC-ESRD, as well as that D-TPL and ND-TPL ESRD are significant prognostic factors of OS and CSS.

In conclusion, this is the first study to present the nationwide prevalence of PC in patients with ESRD (10.1%) in Korea, and the fact that presence of ESRD in patients, irrespective of with or without dialysis or kidney transplantation is a significant survival factor along with other significant clinico-pathological factors. Further investigations on PC-ESRD patients who have undergone kidney transplantation are needed to better understand optimal screening methods to avoid overtreatment and under treatment in this unique and challenging patient population.

## MATERIALS AND METHODS

This study was approved by the Institutional Review Board (IRB) of the National Cancer Center and Cancer Research Institute in Korea (IRB no.: NCC1310250. The database and methodology of cancer statistics have been described in detail elsewhere; additionally, the database has been the source for numerous epidemiological studies [27] that used cancer incidence and mortality data from 2002 to 2012 from the Korean National Health Insurance System of Statistics Korea [27] and the Korea National Cancer Incidence Database of Korean Central Cancer Registry [28].

### Study population

PC diagnosis was based on the International Classification of Diseases, Tenth Edition (ICD-10) diagnosis code (C61) in the Korean Central Cancer Registry between 2003 and 2010. Population data from 2002 to 2011 were obtained from the resident registration population, as reported by Statistics Korea. Due to the time required for data collection, compilation, quality control and analysis, the incidence and mortality data for a specific year are usually available at least 1 year later. Therefore, 38,925 patients with complete medical records, including survival data from the Korean National Health Insurance System until follow-up in 2011, were screened.

The Surveillance, Epidemiology, and End Results (SEER) staging classification was used to stage PC. Diagnostic ICD-10 codes for diabetes (E10.2, E11.2, E12.2, E13.2, and E14.2), renal failure (N17.0, N17.1, N17.2, N17.8, N17, N18, N19, N99.0, T79.5, I13.1, I13.2, I12.0, I12.9, and E87.2), dialysis (Z99.2, Z49.0, Z49.1, Z49.2, N18.5, T85.6, T82.8, T82.4, Y60.2, Y61.2, Y84.1, and E85.3), and kidney transplantation (Z94.0 and T86.1) were used to select 3945 (10.1%) patients with ESRD before they were diagnosed with PC; these subjects were enrolled in the study (ESRD group). Patients with secondary malignancies were excluded from our study.

### Statistical analysis

The Students t-test, chi-square test, and Fisher exact test were used to compare differences between groups. To predict the incidence and mortality of PC, we first performed a joinpoint regression analysis on available data to determine the year when significant changes occurred in cancer trends according to presence and absence of ESRD (e.g., dialysis, renal transplantation, and no dialysis or transplantation). The survival analysis was performed using Kaplan-Meier curves with log-rank tests of 162 (4.1%) patients with dialysis or kidney transplantation (D-TPL group), and 3783 (95.9%) without dialysis and transplantation (ND-TPL group). A competing-risks analysis was adapted in the multivariate analysis for CSS. Two-sided p-values <0.05 were considered statistically significant. All statistical analyses were performed using SAS 9.4 (SAS Institute, Cary, NC, USA).

## Abbreviations

CCI: Charlson comorbity index
PC: prostate cancer
ESRD: end-stage renal disease
PSA: prostate-specific antigen
BMI: body mass index
PC-ESRD: prostate cancer patient with end stage renal disease
SEER: Surveillance, Epidemiology, and End Results
IRB: Institutional Review Board
NIH: National Institutes of Health
D-TPL: patients with dialysis or kidney transplantation
ND-TPL: patients without dialysis and transplantation
ND-TPL ESRD: end-stage renal disease patients without dialysis and transplantation
OS: overall survival
CSS: cancer-specific survival
